# The intensification of anticancer activity of LFM-A13 by erythropoietin as a possible option for inhibition of breast cancer

**DOI:** 10.1080/14756366.2020.1818738

**Published:** 2020-09-10

**Authors:** Dariusz Rozkiewicz, Justyna Magdalena Hermanowicz, Anna Tankiewicz-Kwedlo, Beata Sieklucka, Krystyna Pawlak, Robert Czarnomysy, Krzysztof Bielawski, Arkadiusz Surazynski, Joanna Kalafut, Alicja Przybyszewska, Mariusz Koda, Katarzyna Jakubowska, Adolfo Rivero-Muller, Dariusz Pawlak

**Affiliations:** a Department of Pharmacodynamics, Medical University of Bialystok, Bialystok, Poland; b Department of Clinical Pharmacy, Medical University of Bialystok, Bialystok, Poland; c Department of Monitored Pharmacotherapy, Medical University of Bialystok, Bialystok, Poland; d Department of Synthesis and Technology of Drugs, Medical University of Bialystok, Bialystok, Poland; e Department of Medicinal Chemistry, Medical University of Bialystok, Bialystok, Poland; f Department of Biochemistry and Molecular Biology, Medical University of Lublin, Lublin, Poland; g Department of General Pathomorphology, Medical University of Bialystok, Bialystok, Poland; h Department of Pathomorphology, Comprehensive Cancer Center, Bialystok, Poland

**Keywords:** Erythropoietin, LFM-A13, Bruton’s tyrosine kinase, breast cancer, zebrafish

## Abstract

Recombinant human erythropoietin (Epo) is an effective and convenient treatment for cancer-related anaemia. In our study for the first time, we evaluated the effect of simultaneous use of Epo and Bruton’s tyrosine kinase (BTK) inhibitor LFM-A13 on the viability and tumour development of breast cancer cells. The results demonstrated that Epo significantly intensifies the anticancer activity of LFM-A13 in MCF-7 and MDA-MB-231. The featured therapeutic scheme efficiently blocked the tumour development in zebrafish experimental cancer model. Epo and LFM-A13 administered together resulted in effective cell killing, accompanied by attenuation of the BTK signalling pathways, loss of mitochondrial membrane potential (MMP), accumulation of apoptotic breast cancer cells with externalised PS, a slight increase in phase G0/G1 and a reduction in cyclin D1 expression. Simultaneous use of Epo with LFM-A13 inhibited early stages of tumour progression. This therapeutic scheme may be rationale for further possible research.

## 1. Introduction

Recombinant human erythropoietin (Epo) is an effective and convenient treatment for cancer-related anaemia. The introduction of Epo nearly twenty years ago revolutionised the treatment of this complication. Beside normalisation of haematological parameters (increase in the number of erythrocytes and haemoglobin concentration), Epo leads to improved effectiveness of radiation and chemotherapy, and shortens hospitalisation. Unfortunately, the most of the study results published in the last several years indicate that besides the positive effects on haematological parameters it has the serious adverse effect of promoting the neoplastic process^1^^,^^2^. Chan et al. emphasising an active role of Epo in breast cancer progressi^3^.

New light on the use of Epo in breast cancer has been shed by results published by Zhou who demonstrated that, besides the active role of Epo in breast tumourigenesis, the inhibition of Epo-stimulated intracellular pathways in tumour cells abolishes the neoplastic process^4^. They suggested that JAK2 inhibition in combination with Epo treatment affects lung cancer cells to a much higher extent than erythroid progenitor cells^5^. Moreover inhibition of Epo-induced JAK activation by Fedratinib was synergistic with chemotherapy for breast tumour-initiating cells^4^^,^^5^.

LFM-A13 is first inhibitor of Bruton’s tyrosine kinase (BTK) which decreases tumour progression in the MMTV/Neu transgenic mouse model of HER2 positive breast cancer as effectively as paclitaxel and gemcitabine^6^. It has also proven anti-proliferative and proapoptotic activity against breast and prostate cancer^7–9^. Aalipour and Advani^10^ demonstrated that BTK inhibition blocks NFκB-DNA binding, inhibits DNA synthesis, reduces proliferation, survival and cell migration, interrupts integrin-mediated adhesion to fibronectin, decreases cellular response to tissue homing chemokines (CXCL12, CXCL13, and CCL19), and induces apoptosis^11^^,^^12^. An increasing body of experimental and clinical data in recent years supports a major role of BTK not only in B cell malignancies^13^ but also in other solid tumours, including breast^14^ ovarian^15^ and prostate cancer^8^.

So far no one has studied the effect of co-administration of Epo with compounds effective in breast cancer treatment. The aim of this study was to evaluate the effects of simultaneous use of Epo and LFM-A13 on the viability of breast cancer cells, both MCF-7 and MDA-MB-231 lines, *in vitro* and in zebrafish embryo xenograft model. We examined apoptosis using several biochemical markers, such as phosphatidylserine externalisation, loss of mitochondrial membrane potential (MMP), and expression of caspase 6. We also explored the level of BTK expression in breast cancer samples obtained from patients and the effects of LFM-A13 alone and in combination with Epo on cell cycle progression. The results demonstrated that simultaneous use of Epo and LFM-A13 can strongly induce apoptosis in breast cancer cells. For the first time, we reported an effectiveness of combination of Epo and LFM-A13 in experimental model of breast cancer.

## 2. Materials and methods

### 2.1. Cell cultures

The studies were performed on the oestrogen-dependent MCF-7 (ATCC, HTB-22) and the oestrogen-independent MDA-MB-231 (ATCC, HTB-26) cell lines obtained from the American Type Culture Collection (ATCC, Manassas, VA). Both cell lines were maintained in DMEM without phenol red supplemented with 10% foetal calf serum (Sigma, ‎St. Louis, MO), 50 U/ml penicillin (Sigma, ‎St. Louis, MO), 50 μg/ml streptomycin (Sigma, ‎St. Louis, MO) at 37 °C in a 5% CO_2_ incubator. Cells were generally maintained in 75 cm^2^ flasks (Sarstedt, Newton, NC), but for the experiments cells were plated onto 100-mm dishes (Sarstedt, Newton, NC) with 6 ml of medium. When cells reached 80% confluence, they were collected and seeded in 6-well culture plates (Sarsted, Newton, NC) at a density of 0.25 × 106 cells per well. The cells were incubated for 48 h prior to treatment and without serum in DMEM medium. The control was media with PBS only.

### 2.2. Exogenous erythropoietin and LFM-A13 administration

Breast cancer cell lines MCF-7 and MDA-MB-231 were incubated with exogenous Epo beta at concentrations of 10, 30, and 100 IU/ml, LFM-A13 at concentrations of 10, 30, and 100 µM, and a combination of these drugs (Epo + LFM-A13). Epo^16^ and LFM-A13^17^^,^^18^ concentrations were chosen based on data from the literature. Epo at a concentration of 100 IU/ml is normally used in these types of experiments; it is widely accepted and often used by other research teams^16^^,^^19^. To evaluate whether the antiproliferative effects of drug combinations of Epo with LFM-A13 were synergistic, additive, or antagonistic, drug combinations at several constant ratios were evaluated. For each drug (alone or in combination), three independent experiments were performed. Analysis was done using CompuSyn software (http://www.combosyn.com), which allows for automated simulation of synergism and antagonism at all dose and effect levels using the Chou-Talalay method^20^.

### 2.3. Proliferation assay

Assay was performed according to the method described by Mosmann using 3–(4,5-dimethylthiazol-2-yl)-2,5-diphenyltetrazolium bromide (MTT)^21^. Epo-LFM-A13 interactions were assessed by combination index (CI) analysis using the CompuSyn software program (ComboSyn, Inc., Paramus, NJ) that is based on the Chou-Talalay equation^20^. For drug combinations, cells were treated with three independent concentrations at a constant ratio (1:1). The fraction affected (Fa) was calculated on the basis of MTT measurements of the treated samples. Interpretations of the CI results follow: CI values <1 indicate synergism; =1 indicate additivity; >1 indicate antagonism.

### 2.4. Flow cytometry

Flow cytometry analysis of MMP and assessment of annexin V binding have been described previously^22^. The distribution of the cell cycle phases was analysed using flow cytometry. Briefly, MCF-7 and MDA-MB-231 cells were seeded into 6-well plates at a density of 2.5 × 10^5^ cells per well and treated with Epo at a concentration of 100 IU/ml, LFM-A13 at a concentration of 100 µM, and a combination of these drugs (Epo + LFM-A13) for 24 h. After incubation, the cells were harvested and then fixed with 1 ml of 70% ethanol and stored overnight at −20 °C. Before analysis, the cells were re-suspended in PBS, treated with 50 μg/ml of DNase-free RNase A Solution (Promega, Walldorf, Germany), and stained with 100 μg/ml of PI. The FACSCanto II flow cytometer (BD Bioscences Systems, San Jose, CA) was used to analyse the samples.

### 2.5. Western blot

After stimulation with Epo (100 IU/ml), LFM-A13 (100 µM), and combinations of these agents at 37 °C for 5 min (for phosphorylated BTK), and for 48 h (for BTK, cyclin D1, full length-caspase 6, cleaved-caspase 6), the cells were lysed in NP-40 (50 mM Tris–HCl (pH 8.0), 150 mM NaCl, 1% Triton X-100, and a protease inhibitor cocktail (Roche, Basel, Switzerland)). The lysate was centrifuged at 10,000 × *g* for 20 min at 4 °C. An aliquot (10 µl) of the supernatant was subjected to electrophoresis in a 10% SDS-polyacrylamide gel, followed by transfer to 0.2-μm pore-size nitrocellulose membrane (Bio-Rad, Hercules, CA) according to the method described in the manual accompanying the unit. Blots were blocked for 1 h at room temperature with 5% non-fat milk (Bio-Rad, Hercules, CA) in Tris-buffered saline, pH 8.0 (Sigma-Aldrich, St. Louis, MO). The membrane was incubated with mouse monoclonal clone 53/BTK against BTK (BD Biosciences, San Jose, CA Cat# 611117), rabbit polyclonal Tyr223 antibody against phospho BTK (Cell Signalling Technology, Danvers, MA Cat# 5082 P), rabbit polyclonal antibody against caspase-6 (Cell Signalling Technology, Danvers, MA Cat# 9762), rabbit monoclonal antibody against cyclin D1 (Abcam, Cambridge, UK Cat# ab134175), or mouse monoclonal antibody against beta-actin (Sigma-Aldrich, St. Louis, MO Cat# A2228, RRID:AB_476697) in TBS-T (20 mM Tris–HCl buffer (pH 7.4) containing 150 mM NaCl and 0.05% Tween 20) overnight. Alkaline phosphatase-conjugated secondary goat polyclonal antibody against mouse (Sigma-Aldrich, St. Louis, MO Cat# A3562, RRID:AB_258091) or secondary goat polyclonal antibody against rabbit (Sigma-Aldrich, St. Louis, MO Cat# A3687, RRID:AB_258103) were added at a 1:10,000 dilution in TBS-T and incubated for 1 h with slow shaking. The nitrocellulose was then washed with TBS-T (2 × 10 min) and exposed to the Sigma-Fast BCIP/NBT reagent.

### 2.6. Immunofluorescence

Immunofluorescence and confocal microscopy were used to examine the spindle features of the MCF-7 and MDA-MB-231 cell lines treated with Epo, LFM-A13, and Epo + LFM-A13, as previously described^23^.

### 2.7. Zebrafish husbandry, establishment of xenograft

The zebrafish (*Danio rerio*) were raised at 28.5 °C in E3 buffer with cycles of 14/10 h of light/darkness and fed in accordance with guidelines established by the Research Animals Department of the RSPCA. Breast cancer MCF-7 and MDA-MB-231 cells were labelled with CM-Dil (chloromethylbenz-amino derivatives of 1,1′-dioctadecyl-3,3,3′,3′-tetramethylindocarbocyanine perchlorate) before transplantation. CM-DiI dye stock was prepared as recommended by the manufacturer (Thermo Fisher Scientific Inc., Waltham, MA). Cells were incubated for 20 min at 37 °C in 5 μg/ml CM-Dil solution, washed three times with PBS to remove the unincorporated dye, and the stained cells were re-suspended in PBS at the final concentration of 1 × 10^7^ cells/ml^24–26^. The zebrafish embryo was manually dechorionated 36 h post-fertilisation (hpf) and after another 12 h were anaesthetised by placement in 0.04 mg/ml ethyl 3-aminobenzoate methanesulfonate tricaine which is a water soluble, fast-acting anaesthetic agent. Zebrafish embryos were then transferred to thin film of low-melting-point agarose to stabilise the fish in a lateral position. Labelled breast cancer cells were loaded into a borosilicate glass needle pulled by a P-1000 Next Generation Micropipette Puller (Sutter Instrument Company, Novato, CA). Suspension containing about 100–200 cells into the inferior section of the yolk sac in a single injection by using an electronically regulated air-pressure microinjector (Narishige IM-300 Microinjector). After injection, zebrafish were washed once with fish water and transferred to 6-well plate containing 2 ml of fresh fish water. MCF-7 and and MDA-MB-231-xenografts (72 hpf) were incubated at 33 °C with combination of exogenous Epo beta (100 IU/ml) and LFM-A13 (100 µM) for 48 h. Although embryos are normally allowed to develop at 28.5 °C and human cells at 37 °C, a compromise at 33 °C works well.

### 2.8. Microscope imaging

Living zebrafish embryos were anaesthetised by tricaine and embedded in a lateral orientation. Animals were analysed for cytoplasmic fluorescence intensity. Images of CM-Dil-labelled cells were acquired using an EVOS M5000 Imaging System with the following filters: GFP (470/22 nm Excitation; 510/42 nm Emission) and Texas Red (585/29 nm Excitation; 624/40 nm Emission). Image analysis was performed using ImageJ version 1.51 software (National Institute of Health, Sacaton, AZ).

### 2.9. Patients and tissue samples

The expression of Bruton kinase, HER2, progesterone receptors, oestrogen receptor alpha (ERα), and Ki-67 was assessed in breast cancer samples obtained from 95 women, aged 28–82 years (mean age 59.77 ± 13.16), who underwent partial or total mastectomy and lymph node dissection for primary breast cancer. All tissue samples were fixed in 10% buffered formaldehyde solution, embedded in paraffin blocks at 56 °C and stained with haematoxylin and eosin. Histopathological examination of sections was performed on the basis of the WHO and TNM breast tumour classification^27^. These studies were conducted in accordance with the ethical standards described in the Helsinki Declaration of 1964 and its latest review in 2000 and approved by the Ethics Committee at the Medical University of Bialystok. All patients included in the study signed informed consent forms. The antibodies used in the study were: BTK (Santa Cruz Biotechnology, Dallas, TX sc-81159), Ki-67 (Ventana, Oro Valley, AZ), oestrogen receptor alpha (ER, Clone SP1, Ventana, Oro Valley, AZ), progesterone receptor (PR, Clone 1E2, Ventana, Oro Valley, AZ), and Her-2/neu (Clone 4B5, Ventana, Oro Valley, AZ). A total of 39/75 (52%) of the patients had involved lymph nodes at the time of diagnosis. Our study included invasive carcinomas of no special types, 4 cases in G1 grade, 57 cases in G2 grade, and 20 cases in G3 grade. The analysis was evaluated by two independent pathologists who were blinded to the clinical information. The evaluation of immunostaining for the studied protein was analysed by light microscopy in 10 high power fields (HPF; magnification, ×200–400) and the mean percentage of tumour cells with positive staining was scored. Immunostaining of BTK was classified using a five-point scale: 0 < 10% positive cells; 1 + 10–50% positive cells with weak staining; 2+ >50% positive cells with weak staining; 3+ >50% positive cells with moderate and 4+ >50% positive cells with strong staining. All the procedures were approved by the Local Bioethics Committee (R-I-002/500/2017) and conducted in accordance with institutional guidelines in compliance with national and international laws and guidelines.

### 2.10. Statistical analysis

Shapiro–Wilk’s W test of normality was used for data distribution analysis. In all experiments, the mean values for 4–10 assays ± SD were calculated. Multiple group comparisons were performed using one-way analysis of variance (ANOVA), and significant differences between the groups were assessed using the Tukey–Kramer test or non-parametric Mann–Whitney U-test. In order to evaluate correlations between the studied parameters, the Spearman’s rho correlation test was used. Calculations were performed using GraphPad version 6 Prism software (GraphPad Software, Inc., La Jolla, CA). The differences were deemed statistically significant when *p* < .05. Quantifications of Western blots and measurement of the fluorescence intensity were analysed using Image J 1.50a software.

## 3. Results

### 3.1. Erythropoietin with LFM-A13 decrease cell proliferation and inhibit tumour development

We first tested the impact of different doses of Epo with LFM-A13 on the proliferation in MCF-7 and MDA-MB-231 lines. In both lines, a decrease in cell viability was observed after 48 h incubation with Epo (100 IU/ml) and LFM-A13 (100 µM) together as compared with the control groups, Epo or LFM-A13 alone (Figure 1(A,B)). Therefore, the highest doses were used in further studies. The addition of Epo to LFM-A13 intensified the impact of LFM-A13 on both MCF-7 (Figure 1(A)) and MDA-MB-231 (Figure 1(B)). The results indicate that Epo may act as a chemosensitizer in breast cancer cells, as we have shown previously in the case of colon cancer^23^. A combination of Epo (10–100 IU/ml) with LFM-A13 (10–100 µM) in a constant ratio indicates synergistic activity in MDA-MB-231 cells (Figure 1(D)). In the case of the MCF-7 line, an additive or mostly synergistic interaction was noted (Figure 1(C)). We thus evaluated the effect of the tested combination on a living organism. Red fluorescent labelled human breast cancer cells were implanted into the yolk sac of wild type (WT) zebrafish 48 hpf embryos (Figure 1(E)). Three days after cells injection, solid tumours were established in 100% of MCF-7 and MDA-MB-231 cells implants (*n* = 20). The fish were incubated with Epo + LFM-A13 at the highest doses for 48 h. The control group received the solvent LFM-A13 (10% DMSO/PBS). In both MCF-7 and MDA-MB-23 xenografts we observed significant reduction in tumour development in Epo + LFM-A13 treated group compared with the control (Figure 1(F)). As shown in Figure 1(G), incubation with Epo + LFM-A13 significantly reduced number of cancer cells in both MCF-7 and MDA-MB-231 xenografts as reflected by the drop in fluorescence intensity (87.27 ± 7.32 *vs.* 56.25 ± 9.76 and 92.15 ± 8.74 *vs.* 48.34 ± 9.35, respectively; *p < .05). This effect was more pronounced in MDA-MB-231. It is well known that BTK plays a crucial role in oncogenic signalling and is responsible for the proliferation and survival of cancer cells^13^. To understand the molecular mechanisms of the antiproliferative effect of the simultaneous use of Epo + LFM-A13, we assessed BTK expression in breast cancer cells. The administration of Epo + LFM-A13 resulted in a decrease of BTK expression in both breast cancer lines (Figure 1(H)).

Figure 1.Effect of erythropoietin beta (Epo), LFM-A13 (LFM), and their combined activity on cell viability of MCF-7 (A) MDA-MB-231 (B); *p < .05 (*vs.* Con); ^*p* < .05 (*vs.* Epo); #*p* < .05 (*vs.* LFM-A13. Combination index analysis of erythropoietin (10–100 IU/ml) combined with LFM-A13 (10–100 µM) at a constant ratio in MCF-7 (C), and MDA-MB-231 (D) cells. Synergistic effects are defined as CI < 1, additive effects are CI = 1, and antagonistic effects are CI > 1. Schematic of xenograft assay and analysis of tumour development. Site-specific injection (yolk sac) of CM-Dil (red) breast cancer cells (MCF-7, MDA- MDA-MB-231) into 48 hpf zebrafish embryos and imaging analysis of tumour growth after 48 h incubation with combination of exogenous erythropoietin beta (100 IU/ml) and/or LFM-A13 (100 µM) (E, F). Quantification of total CM-DiI fluorescence by breast cancer cells 3 d after injection; *n* = 4, *p < .05 (G). Immunoblotting analysis for phospho-BTK (pBTK) and total BTK (BTK) in MCF-7 and MDA-MB-231 cells treated with erythropoietin beta (Epo 100 IU/ml), LFM-A13 (LFM 100 µM), and both for 48 h. Samples used for electrophoresis consisted of 20 µg of protein from 6 pooled cell extracts (*n* = 6). Band staining was quantified by densitometry. Bands of phospho-proteins were normalised to respective total proteins. BTK plus pBTK expressions were assessed in a pooled sample of three independent experiments (H).

**Figure:**
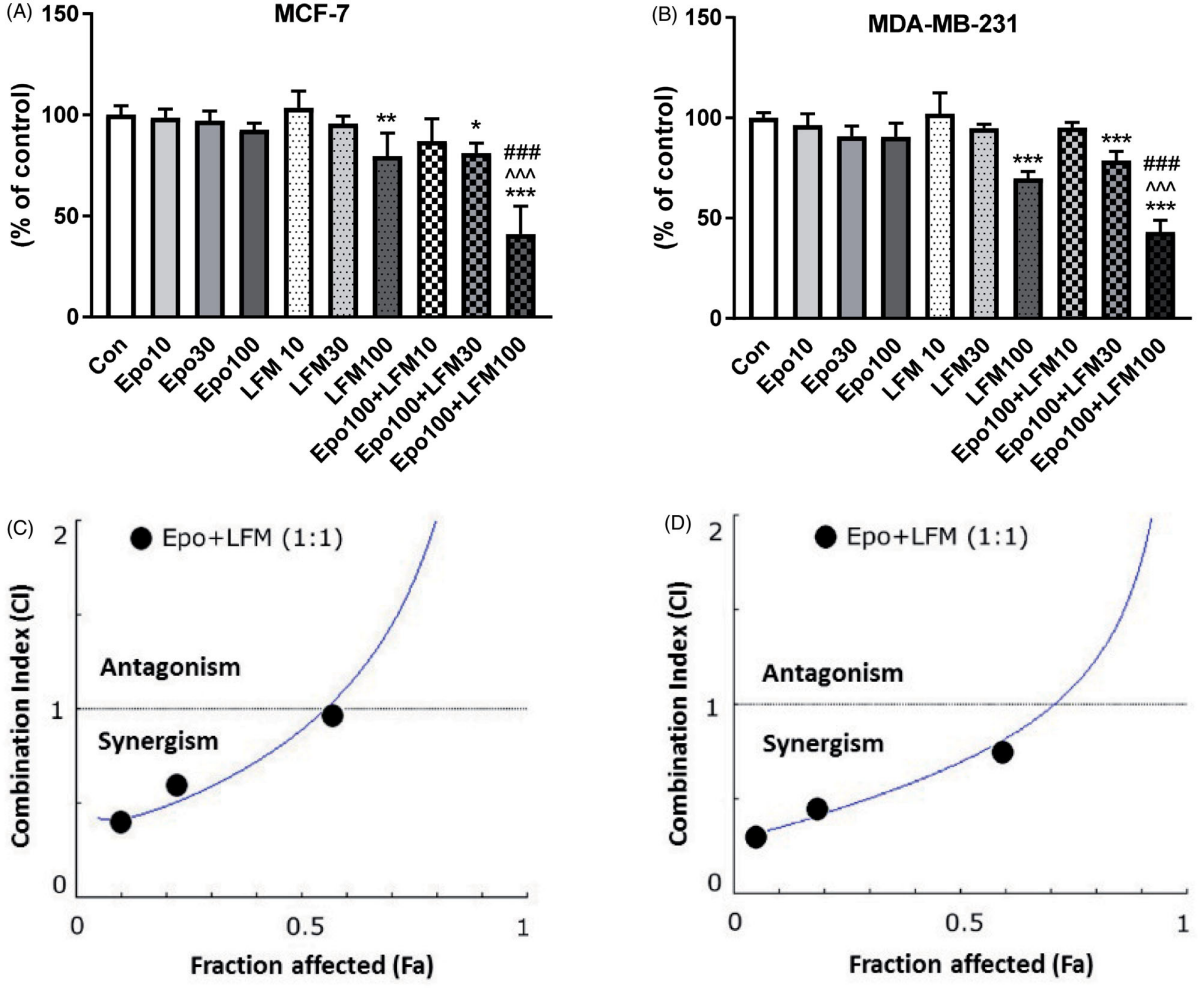

**Figure:**
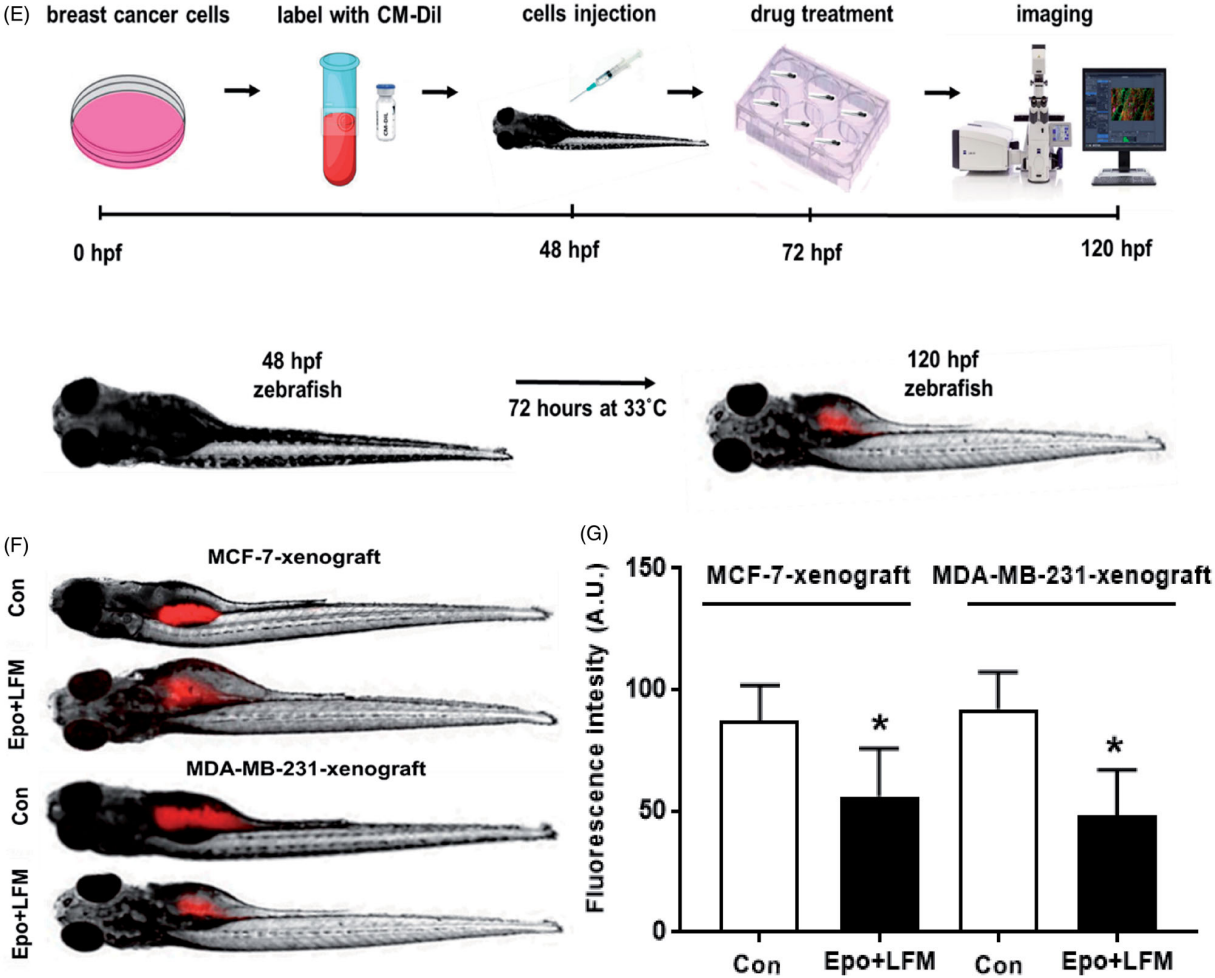

**Figure:**
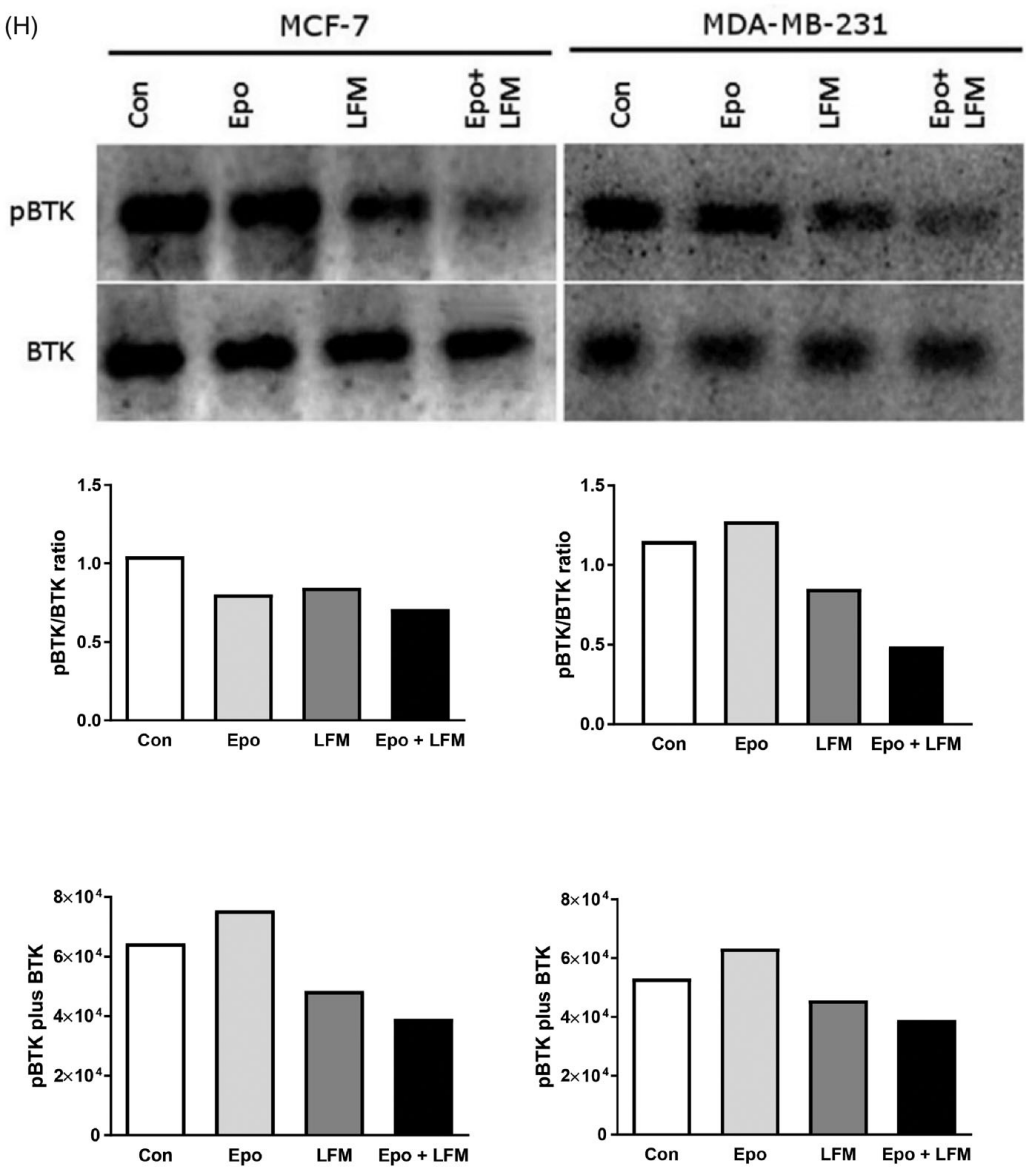

### 3.2. Erythropoietin-LFM-A13 combination enhances apoptosis *via* loss of mitochondrial membrane potential and phosphatidylserine externalisation

**Figure 2.**
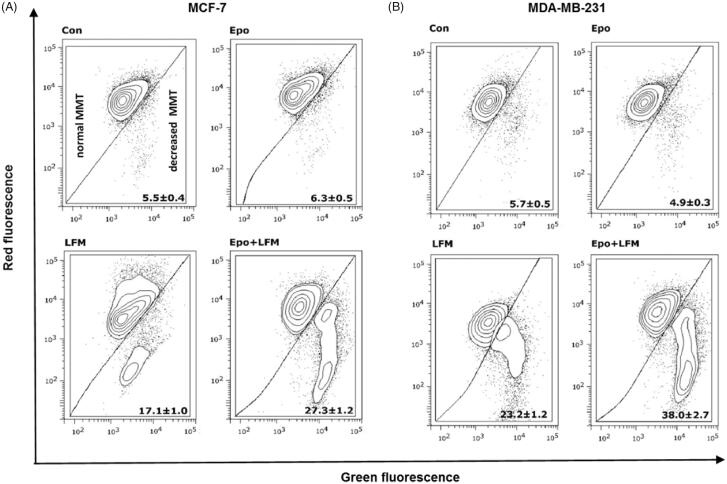
Representative dot-plots presenting the loss of mitochondrial membrane potential (MMP) in MCF-7 (A), and MDA-MB-231 (B) cells incubated with Epo (Epo100, 100 IU/ml) and LFM-A13 (LFM100, 100 μM) for 48 h (mean ± SD; *n* = 3). Cells with normal MMT are shown on the left side of the plots, cells with decreased MMT on the right side of the plots.

Mitochondria have an essential role in apoptosis mediation^28^. It is well known that, at an early stage, apoptotic stimuli alter the mitochondrial transmembrane potential, which can be monitored by fluorescence of the dye JC-1. Therefore, we next wanted to assess the alterations of mitochondrial transmembrane potential, an integral component of cellular energy homeostasis and normal cellular function, by using flow cytometry analysis. We found that Epo + LFM-A13 increased the number of cells with decreased the levels of MMP in both MCF-7 (Figure 2(A)) and MDA-MB-231 (Figure 2(B)) cells at 24 h. We also confirmed that Epo + LFM-A13 induced apoptosis by phosphatidylserine externalisation. It is worth noting that the addition of Epo to LFM-A13 stimulated apoptosis more strongly compared with LFM-A13 alone (Figure 3(A–D)). We also examined whether Epo + LFM-A13 modulates the expression level of caspase-6, a protein that is involved in apoptosis regulation. As shown in Figure 3(E), the administration of LFM-A13 alone led to an increase in the expression of caspase-6. Moreover, the administration of LFM-A13 with Epo caused more strongly upregulated expression of this protein in both tested lines, but especially in the MDA-MB-231 cells. The presented results clearly confirmed an increase in apoptosis induction in breast cancer cells by the combination of Epo + LFM-A13.

Figure 3.Representative flow cytometry dot-plots for Annexin V‐FITC assay in MCF-7 (A) and MDA-MB-231 cells (C) incubated with Epo (100 IU/ml) and LFM-A13 (100 μM) for 48 h (mean ± SD; *n* = 3). The live cells appear at the lower left corner in the plots; the early apoptotic cells appear at the lower right corner; the necrotic cells appear at the upper left corner; and dead cells appear at the upper right corner. The percentage of apoptotic MCF-7 cells (B) incubated with Epo and LFM-A13 is shown in the bar diagram as mean ± SD (*n* = 3). The percentage of apoptotic MDA-MB-231 cells (D) incubated with Epo and LFM-A13 is shown in the bar diagram as mean ± SD (*n* = 3). Immunoblotting analysis for Active caspase 6 and pro-caspase 6 in MCF-7 and MDA-MB-231 cells treated with erythropoietin beta (Epo 100 IU/ml), LFM-A13 (LFM 100 µM), and both for 48 h. Samples used for electrophoresis consisted of 20 µg of protein from 6 pooled cell extracts (*n* = 6). The band staining was quantified by densitometry. Bands of phospho-proteins were normalised to respective total proteins (E).

**Figure:**
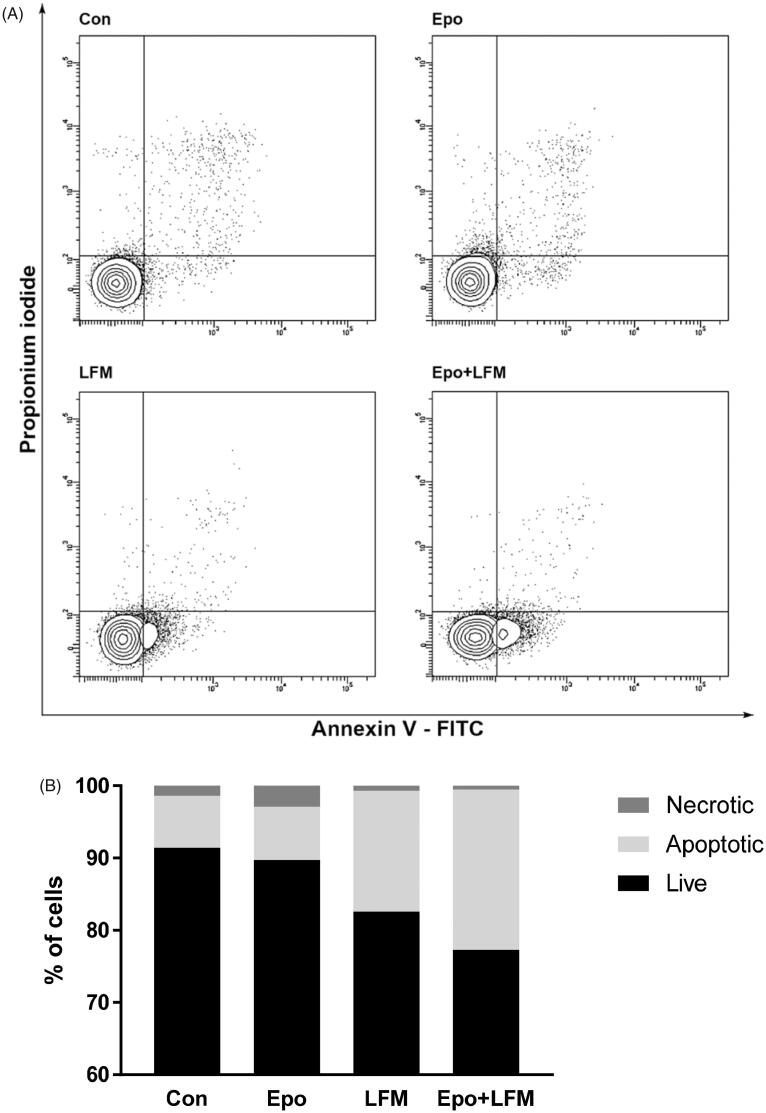

**Figure:**
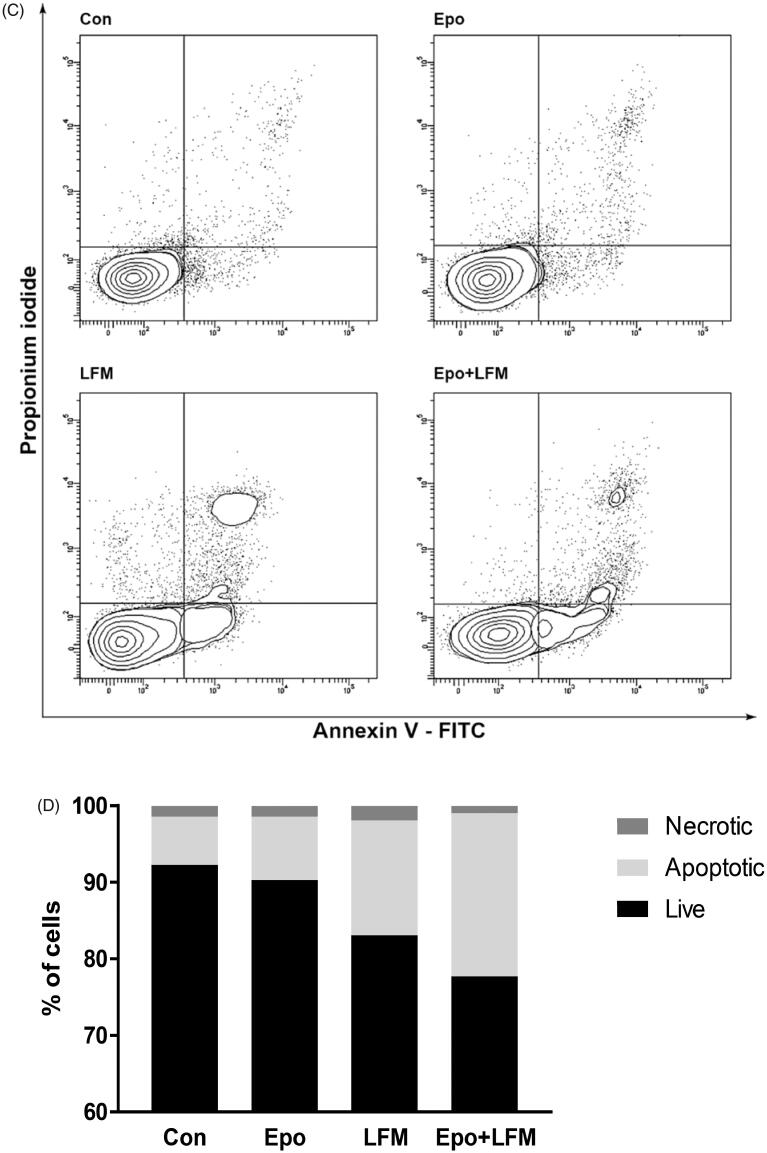

**Figure:**
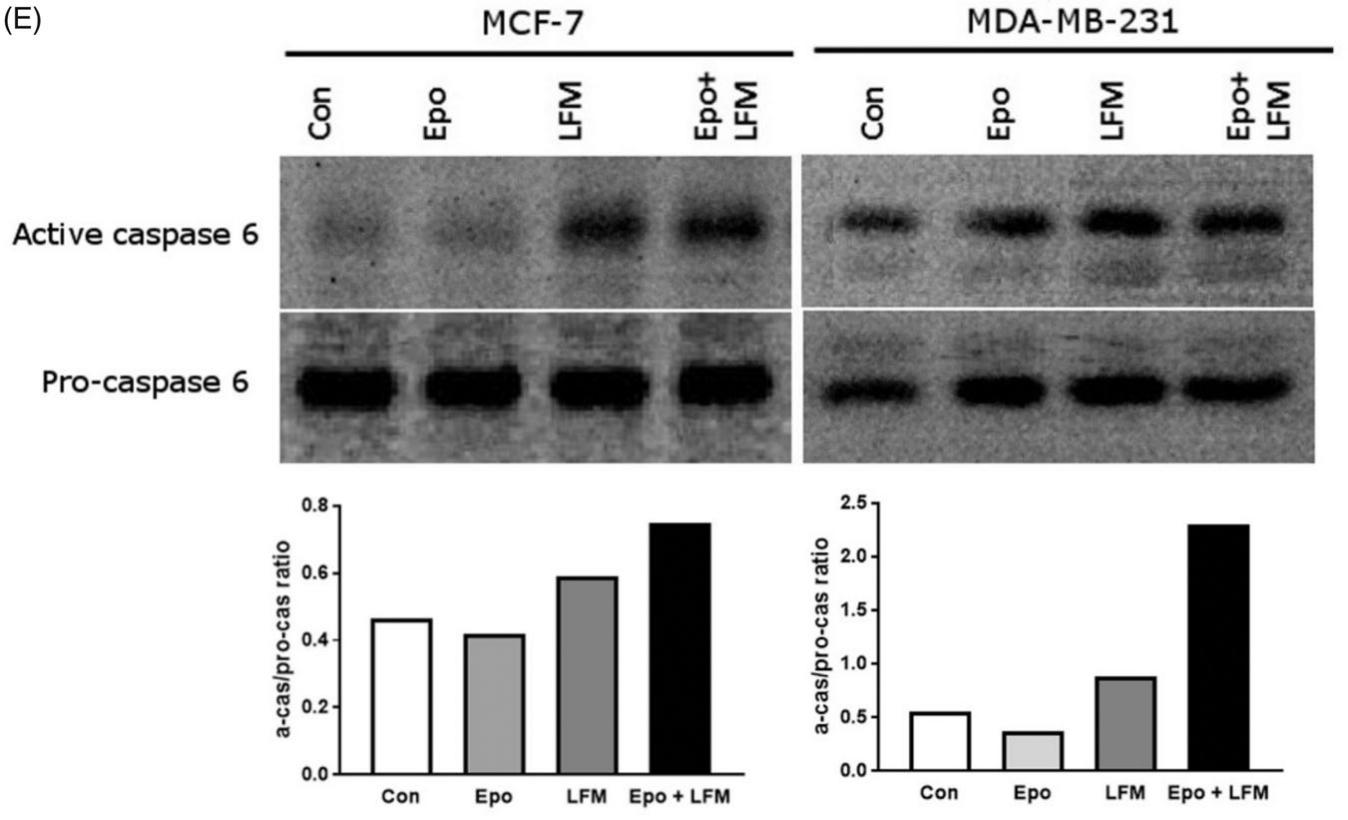

### 3.3. Modulation of cell survival and cell cycle regulator by erythropoietin-LFM-A13 combination in breast cancer cells

Using flow cytometry, we evaluated the effects of LFM-A13 alone and in combination with Epo on cell cycle progression of MCF-7 and MDA-MB-231 cells after 24 h incubation. The G1/S check point is the most critical for the control of cell proliferation *via* the intracellular and extracellular signals related to the transportation and integration of molecules into the nucleus^9^. In MCF-7 and MDA-MB-231 cells, the percentage of S phase fraction was reduced following treatment with Epo + LFM-A13 compared to control (Figure 4(A,B)). This was accompanied by a concomitant increase in the G_0_/G_1_ phase in MCF-7 cells. Cyclin D1 is a member of the highly conserved cyclin proteins that function as controllers in the cell cycle by activating complex with CDK4 and CDK6 to initiate a signalling cascade that directs cell cycle progression^9^. We found Epo + LFM-A13 caused decreased cyclin D1 expression in both lines; however, a stronger effect was observed in the MDA-MB-231 line (Figure 4(C)). The literature data show that down-regulation of cyclin D1 in actively cycling cells significantly increased migration while also decreasing proliferation^29^. Next, using confocal microscopy analysis, we observed that LFM-A13 alone or together with Epo prevented the normal process of microtubule assembly during mitosis. MCF-7 and MDA-MB-231-treated cells developed abnormal monopolar mitotic spindles with highly dense and hyperextended microtubules (Figure 4(D)). Taken together, the observed effect might be also related to the down-regulation of cyclin D1.

Figure 4.Effect of erythropoietin beta (Epo), LFM-A13 (LFM), and their combination on cell cycle progression of MCF-7 (A) and MDA-MB-231 (B). Cells were incubated in the presence of 100 IU/ml of Epo, 100 µM of LFM-A13, and their combination for 24 h at 37̊C, and then examined by DNA flow cytometry, as described in the Methods section. Epo + LFM-A13-treated MCF-7 and MDA-MB-231 cells show S phase fraction reduction and a slight increase in phase G0/G1. Immunoblotting analysis for Cyclin D in MCF-7 and MDA-MB-231 cells treated with erythropoietin beta (Epo 100 IU/ml), LFM-A13 (LFM 100 µM), and both for 48 h. Samples used for electrophoresis consisted of 20 µg of protein from 6 pooled cell extracts (*n* = 6). The band staining was quantified by densitometry. Bands of phospho-proteins were normalised to respective total proteins (C). Erythropoietin beta (Epo), LFM-A13 (LFM), and their combination effect spindle assembly in MCF-7 cells. LFM-A13 and Epo + LFM-A13-treated cells developed abnormal monopolar mitotic spindles and showed aberrant microtubule assembly during mitosis. Orange, γ-Tubulin; Red, α-Tubulin; Blue, DNA/chromosomes (D).

**Figure:**
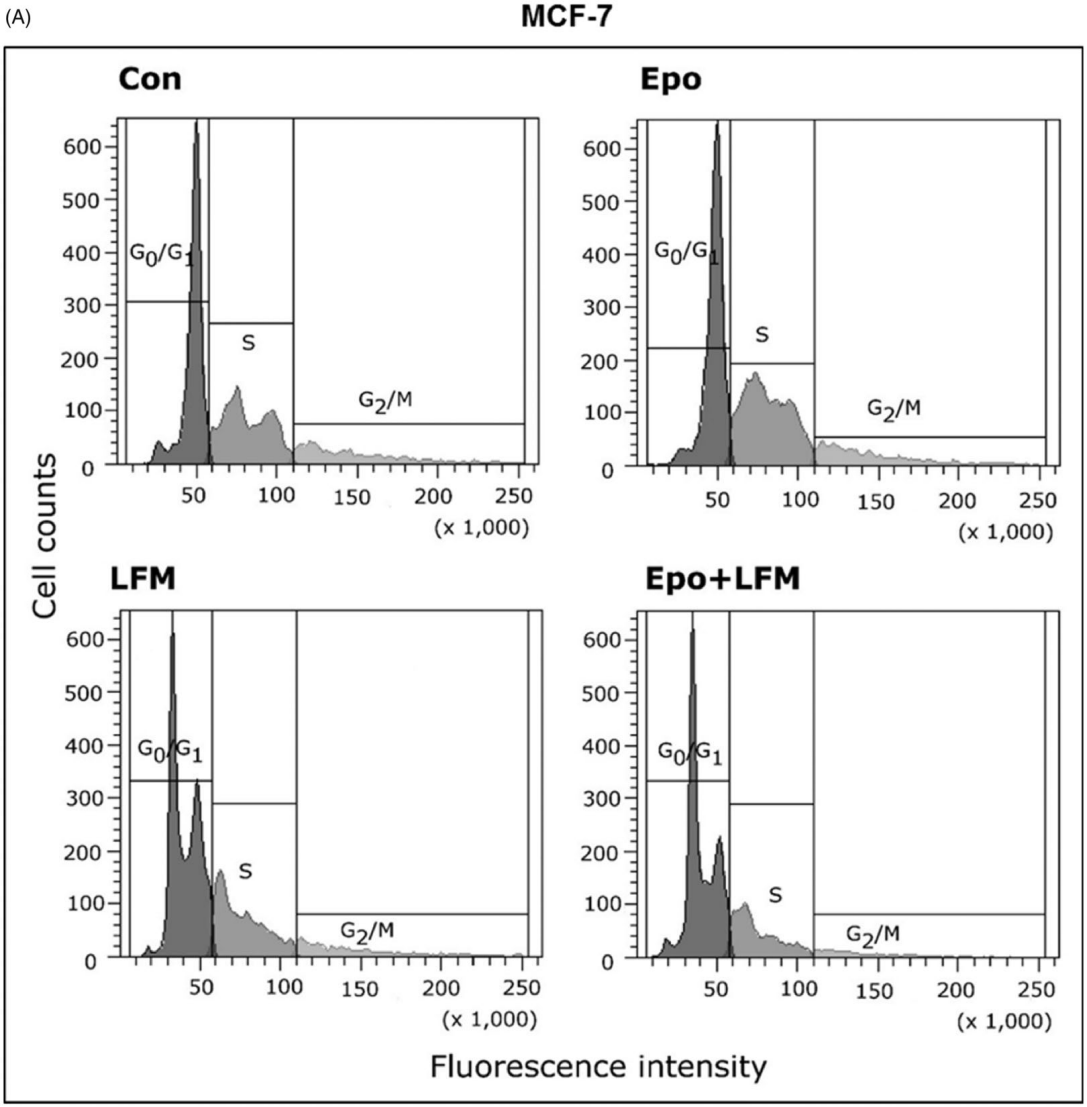

**Figure:**
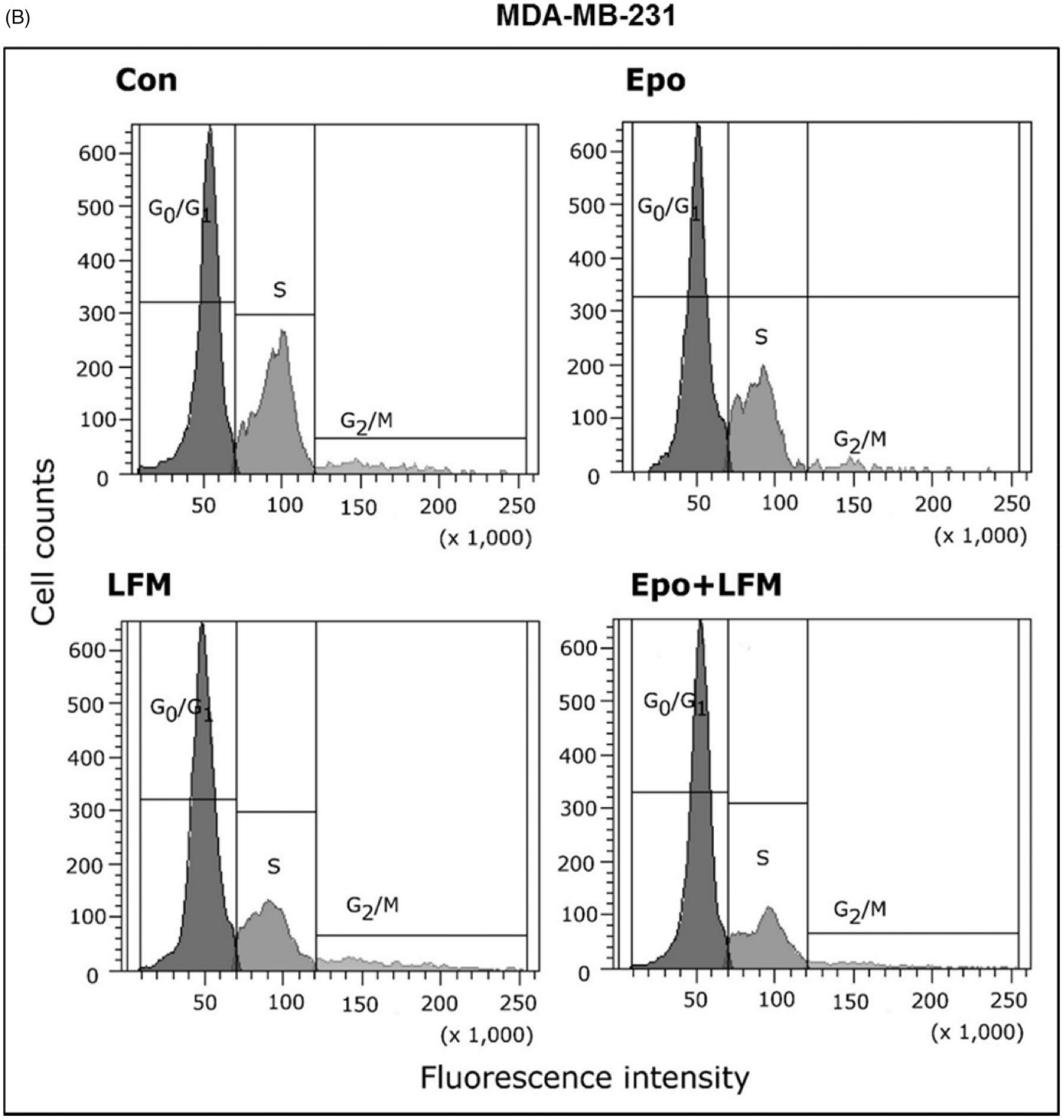

**Figure:**
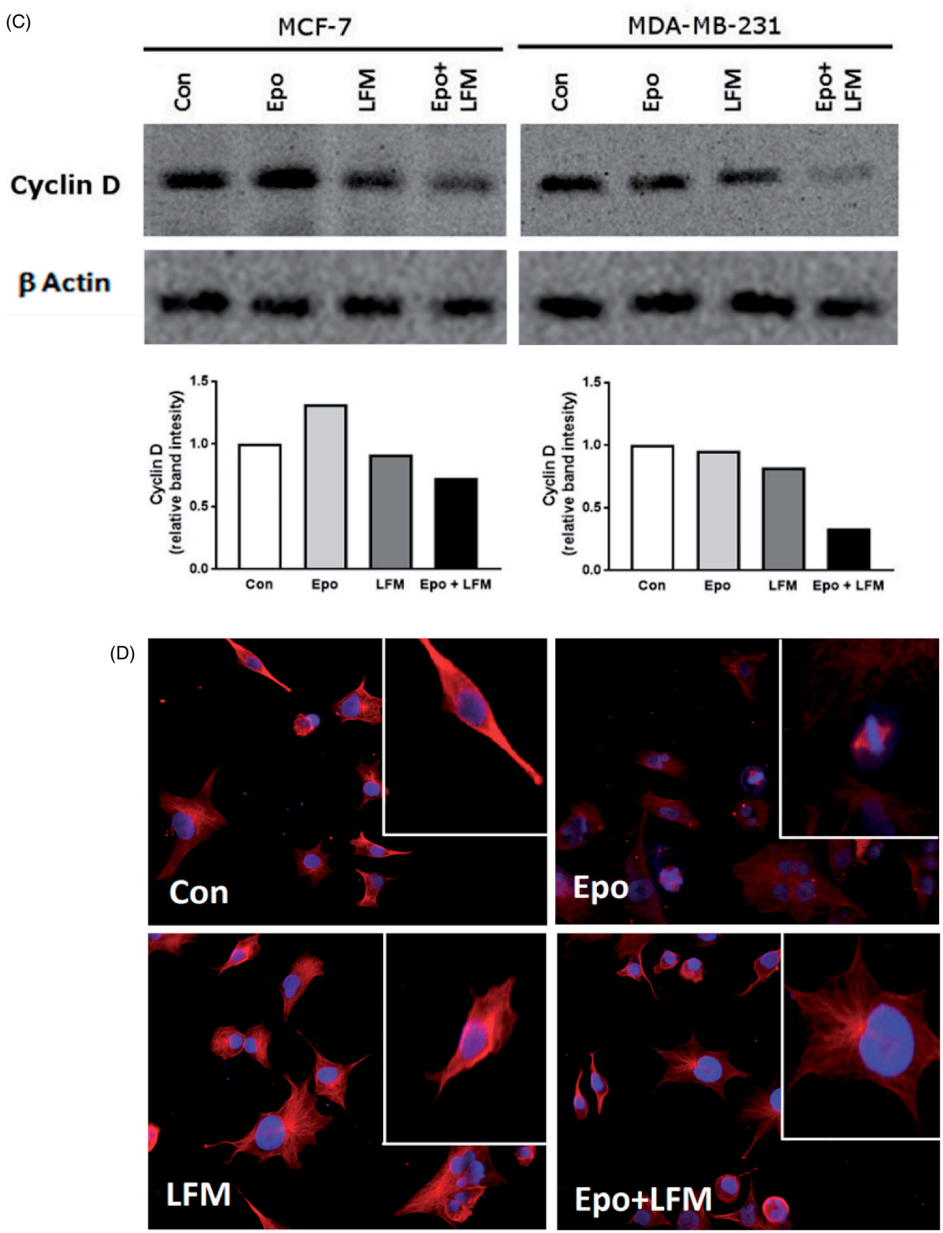

### 3.4. BTK, oestrogen and progesterone receptors, HER2, and Ki-67 expression

**Figure 5.**
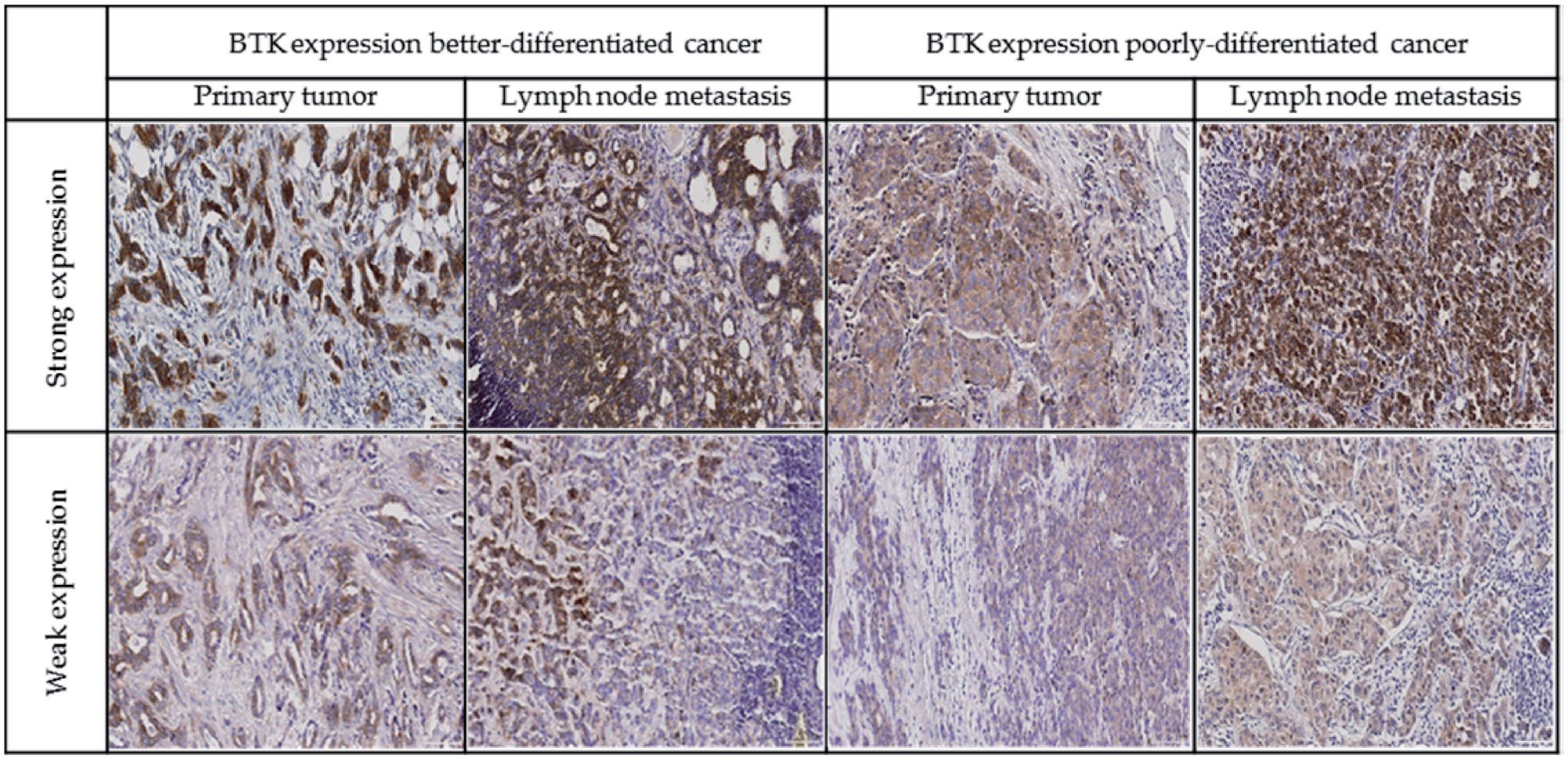
Representative examples of immunohistochemistry for BTK expression in breast cancer cells.

BTK expression was observed mainly in the cytoplasm of the neoplastic cells, but also in a part of cases in nucleus and perinucleus. Fifty-five cases of primary breast cancer out of 90 (61.11%) were positive for BTK (Figure 5). In lymph node metastases BTK expression was found in 23/32 of the cases (71.88%). In better differentiated (G1 and G2) tumours, BTK expression was seen in 35/61 (57.38%) of the specimens. In 11 cases, we observed strong BTK expression. In poorly differentiated (G3) tumours, BTK was observed in 11/20 of the cases (55.00%), four of them with strong BTK expression. Oestrogen and progesterone receptors were expressed in 61/94 (64.89%) and 62/94 (65.96%) of the samples, respectively. In primary tumours, the positive status of HER2 expression was found in 30 patients. To assess whether the expression of BTK undergoes changes during breast cancer progression, this protein was studied in 31 matched pairs of primary tumours and metastases to lymph nodes (Table 1). In 7/31 (22.58%) of the paired cases, BTK expression in primary tumours and metastases to regional lymph nodes was similar. Increased expression of BTK in metastases compared with primary tumours was found in 17/31 (54.84%) of the cases, and decreased expression in 7/31 (22.58%) of the cases. BTK-positive primary tumours developed BTK-negative metastases to regional lymph nodes in 3/23 (13.04%) of the cases. On the other hand, 3/8 (37.50%) of BTK-negative primary tumours produced BTK- positive metastases (Table 1). Spearman’s rho correlation between BTK level in the tumour and node metastasis and parameters such as age, G, pT, HER2, oestrogen, and progesterone receptors, Ki-67 did not show a significant association (Table 2).

**Table 1.** BTK expression in primary tumours and metastases to lymph nodes.

	BTK expression	Primary tumour					
		0	1	2	3	4	Total number of cases
Lymph node metastasis	0	5	1	–	2	–	8
	1	2	1	–	2	2	7
	2	1	3	–	–	–	4
	3	–	7	1	–	–	8
	4	–	3	–	–	1	4
	Total number of cases	8	15	1	4	3	31

Data are expressed as the number of paired cases in each group. BTK expression levels in primary tumours and metastases were assigned on a 0–4+ scale, as described in Materials and Methods.

**Table 2.** Spearman’s rho correlation between BTK level in tumour and node metastasis and selected parameters.

Spearman’s rho	Bruton kinase in primary tumour	Bruton kinase in lymph node metastasis
Bruton kinase in primary tumour		
Correlation coefficient	1	0.308
Sig. (2-tailed)	–	0.092
*N*	90	31
Age		
Correlation coefficient	−0.036	0.225
Sig. (2-tailed)	0.739	0.224
*N*	89	31
G		
Correlation coefficient	0.025	−0.14
Sig. (2-tailed)	0.832	0.505
*N*	76	25
pT		
Correlation coefficient	0.056	−0.023
Sig. (2-tailed)	0.616	0.907
N	83	28
pN		
Correlation coefficient	0.212	–
Sig. (2-tailed)	0.076	–
*N*	71	32
HER2		
Correlation coefficient	−0.143	−0.216
Sig. (2-tailed)	0.181	0.235
*N*	89	32
Oestrogen receptors		
Correlation coefficient	−0.191	0.171
Sig. (2-tailed)	0.073	0.35
*N*	89	32
Progesterone receptors		
Correlation coefficient	−0.112	0.02
Sig. (2-tailed)	0.298	0.915
N	89	32
Ki-67		
Correlation coefficient	−0.102	0.013
Sig. (2-tai)	0.66	0.974
*N*	21	9

## 4. Discussion

Recently, we discovered that adding Epo to LFM-A13 significantly intensified the anticancer action of LFM-A13 in colon cancer both in *in vitro* and *in vivo* conditions^23^. Herein we provide evidence that this combined treatment is also effective against breast cancer. Simultaneous use of Epo with LFM-A13 exhibited antiproliferative activity against both oestrogen-dependent MCF-7 and oestrogen-independent MDA-MB-231 breast cancer cells. The putative mechanism of cytotoxicity is related with an increasing apoptotic response to the applied therapy.

As a first step in exploring the potential implication of the proposed combined therapy, we assessed cell growth to evaluate the effect of simultaneous use of Epo and LFM-A13 on breast cancer cells. Numerous data clearly indicate that LFM-A13 exhibits antiproliferative activity. Inhibition of BTK by LFM-A13 induced growth reduction in chronic lymphocytic leukaemias, diffuse large B-cell lymphomas of the activated B-cell type, and mantle cell lymphomas^30^. Uckun et al. also demonstrated that high doses of this agent possess antiproliferative activity with blocking cell division in a zebrafish embryo model at the 16-cell stage of embryonic development^6^. It has also been shown that the inhibition of BTK expression by LFM-A13 decreased the proliferation of prostate cancer cells, but not normal prostate epithelial cells, which are characterised by low BTK expression^8^. In this study, we not only confirmed the antiproliferative action of LFM-13 in both breast cancer cells lines but also indicated that the addition of Epo to this system further reduced viability of these cells. These results are consistent with our previous findings, which demonstrated that Epo appears to be a chemosensitizer and showed a synergistic antiproliferative effect with LFM-A13 in two colon cancer cells DLD-1 and HT-29^23^.

The high effectiveness of the combination used has also been confirmed in zebrafish embryo xenograft model. In recent years, zebrafish has been recognised as a key model organism for oncological research. It possesses unique advantages: small size, ease of breeding, large numbers of progeny (high confidence in statistical analysis), transparency of zebrafish embryos, and high grade of similarity^24^. Moreover, it is a viable alternative to the use higher-order animals (mice, rats, etc.). In this study, we have established a xenograft model in zebrafish by implanting human tumour cells for 48 h before treatment. We observed a significant antiproliferative effect exerted by the combination of Epo + LFM-A13 in this model.

**Figure 6.**
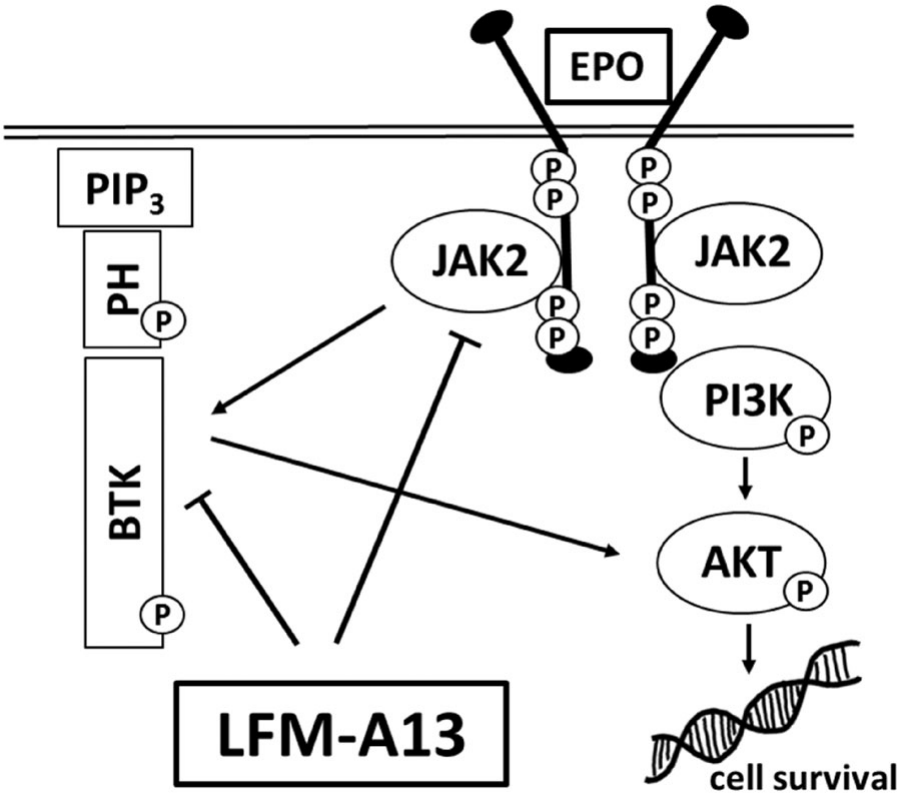
Schematic diagram of intracellular proteins BTK and JAK2 interaction and LFM-A13 mechanism of action. EPO: erythropoietin, LFM-A13: Bruton’s tyrosine kinase inhibitor; JAK2: non-receptor tyrosine kinase; BTK: Bruton’s tyrosine kinase; Akt: protein kinase B; PIP3: phosphatidylinositol-3,4,5-triphosphate; PH: pleckstrin homology domain.

BTK is responsible for the proliferation and survival of cancer cells^13^, it plays a key role in solid tumour development, including ovarian^25^, prostate^8^, and colon cancer^23^. It was also shown that BTK is required for efficient signal transduction by the ligand-activated EpoR. LFM-A13, beside BTK inhibition, was proved to block also Janus kinases (JAK2) binding to EpoR thus breaking the intracellular signal transduction pathway. The data suggest that LFM-A13 directly inhibits BTK and JAK2 kinase activity^31^ and both proteins interact with each other as we shown in Figure 6. Wang et al. reported that this kinase is frequently expressed in human breast cancer cells and tissues^14^. In our study, the presence of BTK in both MCF-7 and MDA-MB-231 cell lines as well as in primary and metastatic breast cancer tissues has been confirmed. Interestingly, both BTK-positive and BTK-negative primary tumours were found to produce BTK-positive as well as BTK-negative metastases.

The differences in BTK expression observed between a primary cancer and related metastases in the same patient or in different patients bearing breast cancer may indicate the presence of a tumour heterogeneity, which can strongly influence the choice of the more appropriate clinical management^32^. Tumour heterogeneity mainly depends on the presence of several different parameters, which can differ from patient to patients, namely hormone receptors expression, HER2 expression or degree of tumour differentiation but also in areas of the same tumour, also during its progression. As has been presented in Table 2, we tried to establish the association between BTK expression and the routinely determined parameters associated with breast cancer but we not able to find such associations. In this study, we showed that LFM-A13 inhibits BTK levels and pBTK/BTK ratio, especially in MDA-MB-231 cells, and co-administration Epo significantly potentiates this effect. This finding indicated that reduced breast cancer cells viability may result from BTK inhibition. We suppose that the potential mechanism of the observed effect may be related to increased pBTK expression in response to Epo, which resulted in enhancing the antiproliferative action of LFM-A13 through the elevation of the number of its target proteins. BTK can mediate downstream signalling to regulate cell differentiation, growth, and apoptosis, whereas LFM-A13 inhibits the BTK-dependent activation of the PI3K/Akt signalling pathway, which is one of the strongest prosurvival intracellular signalling systems^33^. In the context of above observations, simultaneous use of Epo and LFM-A13 seems to be promising option for further investigation of this scheme against breast cancer.

BTK has been classified as a gene whose expression protects breast cancer cells from apoptosis^34^. Using DNA microarrays, Kokabee and collaborators demonstrated that the overexpression of BTK-C isoform is responsible for elevated expression of genes controlling functions of cytoskeletal structure, cell adhesion, and the extracellular matrix, while inhibiting BTK enhanced expression of apoptosis-related genes^7^. Wang et al. noted that ibrutinib increased apoptosis in BT474 and MCF-7 breast cancer cells *via* reduction of BTK phosphorylation and enhancement of cleaved caspase-3^14^. LFM-A13 was also shown to increase apoptosis levels in breast cancer cells^14^. Our results are in line with these observations revealed that LFM-A13 increased the number of MCF-7 and MDA-MB-231 apoptotic cells. Epo with LFM-A13 further enhanced these effects and induced a high level of apoptosis in both of the cell lines used; however, a stronger effect was observed in triple-negative breast cancer cell line (MDA-MB-231). This is an important clinical finding because triple-negative breast cancers (TNBC) are commonly presented with aggressive biological behaviours and often have poor prognosis. The greater sensitivity of this TNBC line was also noticed by us in zebrafish xenografts model.

During apoptosis, the MMP is reduced and it is an early and already irreversible stage of this process. We observed that simultaneous use of Epo with LFM-A13 led to significantly decrease in MMP both in MCF-7 and MDA-MB-231 cells lines. To explain the mechanism how the applied combination is able to induce apoptosis, we examined the expression of caspase-6, which is categorised as an apoptotic effector related with nuclear shrinkage and the direct activator of caspase-8 in the cytochrome c-induced apoptosis pathway^35^^,^^36^. We found that apoptosis induced by LFM-A13 was associated with caspase 6 activation. Moreover, adding Epo to LFM-A13 more effectively upregulated the expression of active caspase-6 compared with LFM-A13 used alone.

A fundamental aspect of tumourigenesis is a dysregulated cell cycle control. We assessed the effects of LFM-A13 alone and in combination with Epo on cell cycle progression of MCF-7 and MDA-MB-231 cells lines. In both breast cancer cell lines, Epo + LFM-A13 treatment resulted in S phase fraction reduction with a significant 40 and 45% decrease in the number of cells in MCF-7 and MDA-MB-231, respectively. At the same time, we noticed a slight increase in phase G_0_/G_1_. The observed effect was associated with a decrease in the expression of cyclin D1, an important regulator of cell cycle progression^37^. Cyclin D1 promotes physiological cell proliferation downstream of mitogens and other extracellular stimuli as G_1_ phase regulatory protein. It was previously demonstrated that the overexpression of cyclin D1 was linked to the development and progression of several cancers including bladder, lung, and breast^38^. A number of anti-cancer agents have been shown to induce cyclin D1 degradation in *in vitro* conditions^37^^,^^39^^,^^40^. Our results are in line with the study of Wang et al., who showed that the BTK inhibitor, ibrutinib, led to an appreciable G_1_-S arrest in HER2 overexpressing BLT474 human breast cancer cells. This cell-cycle delay was correlated with a decrease in cyclin D1 expression^14^.

A key component of normal cell division is the accurate distribution of cellular components, particularly chromosomes, and during mitosis. Chromosomes direct the formation of bipolar mitotic spindles that ensure the equal segregation of chromosomes between daughter cells^41^. Herein, we demonstrated that simultaneous use of Epo with LFM-A13 prevented the normal process of microtubule assembly which led to mitotic aberrations in MCF-7 and MDA-MB-231. Treated cells developed abnormal monopolar mitotic spindles with highly dense and hyperextended microtubules. The results obtained by Kumar and Kim, who showed that LFM-A13 inhibits bipolar spindle assembly formation in human cancer cell lines, including breast cancer and glioblastoma, are in accordance with our^41^.

## 5. Conclusion

The outcomes of this study show that BTK inhibition in combination with Epo may be beneficial and desirable, as this therapeutic scheme effectively inhibit tumour growth *via* enhancement of apoptosis. Our results provide promising but still preliminary material for further preclinical breast cancer study.
